# Learning from the best: How medical-students construct role models in general practice during the COVID-19 pandemic and what factors influence this process, a qualitative study.

**DOI:** 10.12688/mep.20594.2

**Published:** 2025-03-27

**Authors:** Hamish Sutcliffe, Patrick Odonnell, Jane Andrews

**Affiliations:** 1 WMS, University of Warwick Warwick Medical School, Coventry, England, UK; 2 WMG, Coventry, England, UK; 3 Medical Education, University of Dundee Centre for Medical Education, Dundee, Scotland, UK

**Keywords:** Keywords: role-model*; role model; general practice; GP; medical student MESH: Students, Medical, Grounded Theory, General Practice

## Abstract

**Background:**

Role-modelling has been found to strongly influence speciality choice for medical students either positively or negatively. There is a deficit in recruitment toward general practice, set to exacerbate the shortfall in GP numbers over the coming decade in the face of spiralling demand. In medical school, students acquire knowledge, skills and start to form their professional identity by observation and interaction with medical educators through the process of role-modelling. Given the significance of this process, the present study attempted to explore the “lived experience” of medical students encountering potential role models during their GP placement using a qualitative method.

**Methods:**

Following a design based upon the principles of Grounded Theory 10 qualitative interviews were conducted with third-year medical student volunteers at Warwick Medical School. Interviews were recorded, transcribed and analysed using theoretical axial coding demonstrating data saturation in key themes.

**Results:**

Analysis of data gave insights regarding student perception of positive and negative role modelling in three corresponding domains: Personal Attributes, Student Relationship and Patient Relationships.

**Conclusions:**

The findings offer unique insights into the influence and impact of GP role-modelling on medical student’s experiences and perceptions during a time of the Covid-19 pandemic and the immediate post-pandemic period and add to the wider body of literature by exploring the influences GP role-modelling has on medical student training experience. The findings support easily implementable recommendations to strengthen positive role modelling in the GP medical student placement context.

## Introduction and literature review

The NHS Workforce Plan identifies the shortfall of GPs will grow to 15,000 by 2036 without significant change ( *NHS Longterm Workforce Plan*, 2023). In examining the extant literature in the area of role-models within general practice, much attention is given to the positive and negative attributes and behaviours displayed by role-models and the subsequent impacts of this on mentees. Passi *et al.* ( Passi *et al.*, 2013) broadly conceptualise positive doctor role-modelling thematically; identifying ‘clinical competence’, ‘humanistic personal attributes’ (including empathy, respect and compassion) and ‘teaching ability’ as key features of a good mentor. Others, such as Jochemsen ( Jochemsen-Van Der Leeuw *et al.*, 2013) expand on this and point to three dominant positive role-model themes which they referred to as the: “three H’s” of “Heart” (Personal attributes of humility, integrity, collegiality Empathy), “Hands on” (Clinical skill and expertise)” and “Head” (Stimulating critical thinking, being cognisant of ensuring safe and supportive learning environment).

In building on these competence-based definitions Holden *et al*. ( Holden *et al.*, 2020) argued that role-modelling represents an understated component of the General Practice Teaching Triad, with the other two components, programmed teaching and structured experience, gaining far greater attention. Introducing the term “role-modelling consciousness”, Holden *et al*. argue that those recognised as positive role-models are purposefully cognisant of the behaviours they model and project to others. They also identify several significant indicators and themes framing positive role-modelling, including ‘ *personal commitment to excellence and integrity*’ combined with the ‘ *aspiration to create constructive rapport with medical students*’ ( Holden *et al.*, 2020). It is this approach that resonated strongly with the study presented in this paper.

Widely examining the literature, Lamb *et al*. ( Lamb *et al.*, 2022) conducted a Systematic Review of 46 papers from 14 countries and highlighted the importance of medical students (potential future GPs) being exposed to positive GP role-models in shaping and influencing perceptions and later career choices. Lamb *et al.* (2022) observe how secondary care role-models can routinely ‘bash’ general practice: *‘Students perceived the job of a GP as ‘difficult to do well’ as they witnessed secondary care role models ‘bashing GPs’ and their ‘poor’ or ‘inappropriate referrals.’ (pg 22)* negatively impacting medical students’ attitudes before being exposed to General Practice. Emphasising the need to address such negative attitudes, Lamb *et al.* (2022) continue to draw attention both to the value of medical students being exposed to positive role-modelling, and to the detrimental impact of negative role-modelling by doctors in both General Practice and Secondary Care. An obvious consequence of which, is that future doctors are steered away from careers in General Practice.

**Figure 1.**
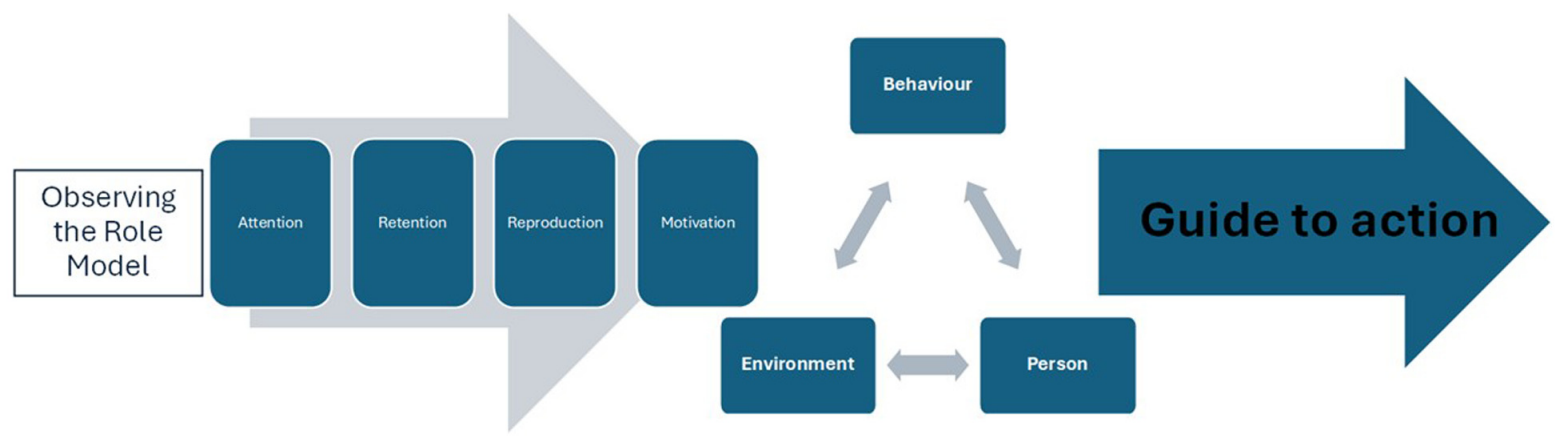
Model of the Observational Process of learning as Applied to Role Modelling by the Clinical Trainer, (Drawn from the work of Bandura (1977) ( Jochemsen-Van Der Leeuw *et al.*, 2013).

It is interesting to note that Bandura’s ( Bandura, 1977) Social Learning Theory has been regularly cited in the literature as providing a useful starting point when considering the influence and impact of role-modelling. Social Learning Theory posits that trainees learn essential professional competencies through a linear process of observation, modelling, and imitation of behaviours, attitudes, and emotional reactions of role-model professionals. From this perspective, learning through role-modelling is conceptualised through four processes: ‘attention’, ‘retention’, ‘reproduction’ and ‘motivation’. In interpreting this to the context of how medical students learn from role-modelling, Figure 1 shows the complex nature of relationships, individual behaviours and learning whereby observed behaviours, progress through a process of reinforcement and imitation to inform future actions. 

**Figure 2.**
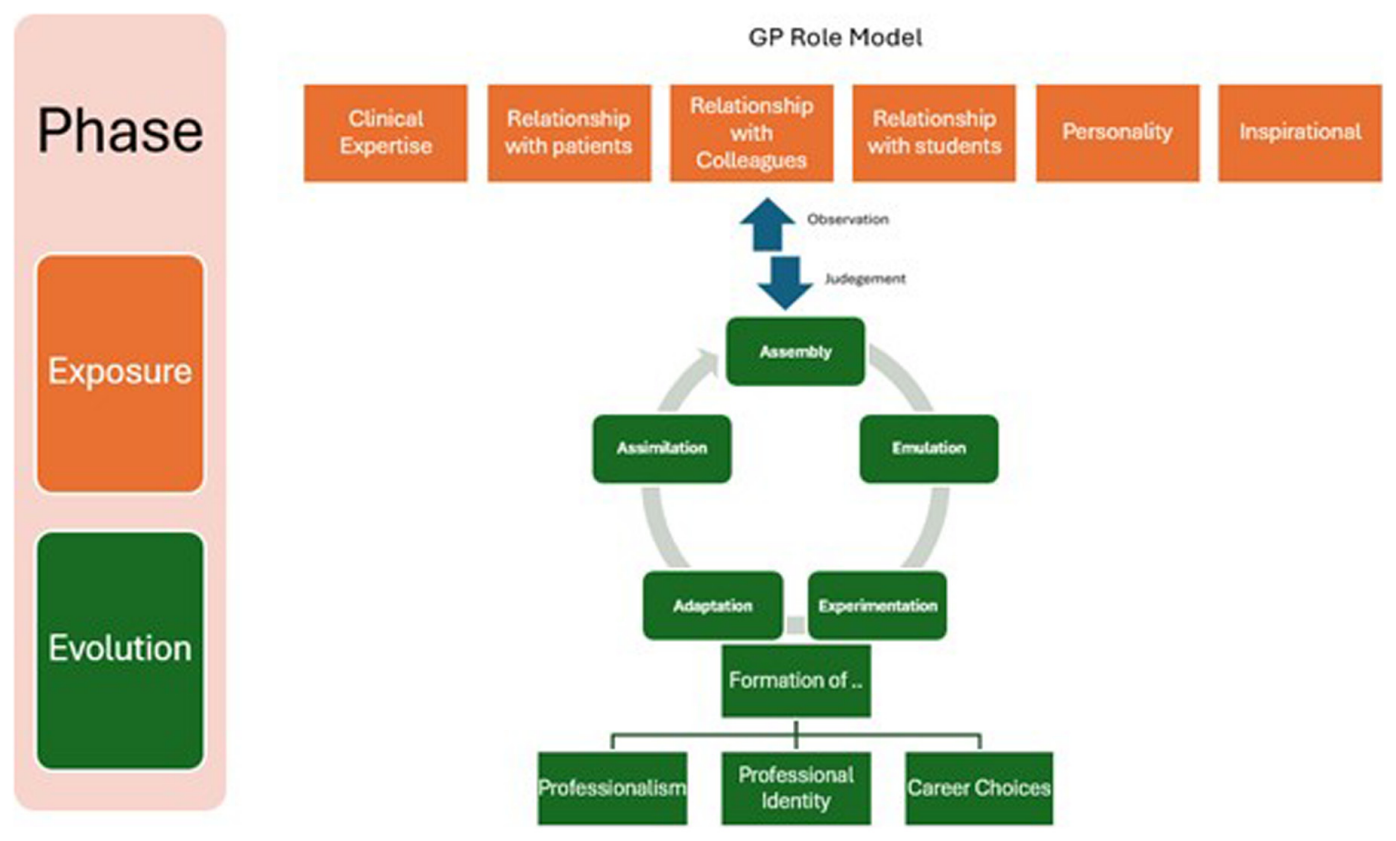
The phases of role modelling, drawing from the work of Passi ( Passi & Johnson, 2016a).

Taking a lead from Social Constructivist theory, Passi and Johnson ( Passi & Johnson, 2016a) expand the principles of behaviourism to posit that the role-modelling process in medicine can be divided into what is termed as ‘Exposure Phase’ (depicted here in light blue in the Figure 2) and ‘Evolution Phase’ (Vertical Column Figure 2).

Applying the Exposure Phase as depicted above in Figure 2 to medical education, role-modeling is most influential when a student becomes embedded in medical practice, working alongside the GP role-model. Building on this, the Evolution Phase involves the medical student observing, (consciously and subconsciously) scrutinizing and processing their perceptions and experiences; filtering out and making judgments on what they perceive as positive values, attributes, and attitudes to emulate going forwards. This evolution phase comprises observation, reflection, internal dialogue and negotiation which ultimately leads to the medical student constructing what they perceive as the ‘ideal professional doctor role-model’ to look up to and follow ( Passi & Johnson, 2016b).

In addition to the importance of role-modelling positive behaviors and building good professional relationships, the nurturing of clinical competencies within an open and professional environment is also identified as being key to the development of future General Practitioners ( Burgess *et al.*, 2015). Whilst there is little doubt that role-modelling has a significant part to play at all stages of medical education, there exists a gap in our understanding of the complex contextual interactions related to role-modelling within the primary care context. There is a need for further qualitative based investigation focusing specifically on questions around the ‘lived experience of medial students in experiencing role-modelling within General Practice’. It is this gap that this paper addresses.

## Methods

Qualitative interviews (n=10) were conducted with third year medical students enrolled at Warwick Medical School. This approach engendered the level of flexibility required to conduct research within the field of medical education. The interview schedule was divided into two main themes, namely perceptions of the negative and the positive attributes of general practice role-models, and a voluntary sampling approach adopted. The benefit of the approach is that it afforded the researcher the opportunity to expand on certain points whilst giving participants the freedom and opportunity to fully describe their experiences and feelings in depth ( Flick, 1998; King, 1994). Interviews were recorded then transcribed, and data analysed using theoretical and axial coding ( Glaser & Strauss, 1967). Whilst the sample size is relatively small, theoretical saturation was achieved in many of the areas discussed.

The data gathering and analysis was informed by Constructivist Grounded Theory (CGT); an approach specifically designed and utilised for investigating and teasing out how people create meaning during social interactions - how they define social settings, and how they construct and present themselves and others with these social settings. It is important to note that CGT differs from other earlier versions of grounded theory because it acknowledges and situates the role of the researcher within a web of significance.

Earlier version of grounded theory ( Glaser & Strauss, 1967) asserted that the researcher can and should be neutral, detached and set aside existing knowledge and understandings about the subject under investigation. However, since its first appearance in the late 1960s grounded theory has evolved and been revised and refined. As such, CGT (advanced by Charmaz, ( Charmaz, 2008) and others such as ( Lindqvist & Forsberg, 2023) recognises the complexities and difficulties associated with the notion of the researcher as temporarily positioning themselves as the neutral and impartial observers. As Lindqvist & Forsberg, (2023: 200) point out under CGT: ‘the researcher is not positioned as being able to put their own knowledge aside and view themselves as a tabula rasa’. Rather for these commentators, under CGT the researcher enters the research process with certain values, preconceptions and preference and as such, they are cast in the role of co-constructor of the emerging data. Thus, the argument unfolding here that we need to be sceptical of any claims that the researcher can easily escape from their own experiences and preconceived ideas and adopt the role of a dispassionate, neutral observer (who remains separate from the participants realities and experiences) able to examine the participants world as an outsider.

Consequently, Charmaz (2008) argues that the researcher must adopt a reflexive stance on their positionality, they must engage with how their own experiences and understanding may shape the research process. Thus, considering wider role of the lead author in this study here (Associate Professor with intimate knowledge and understanding of primary care) CGT is highly relevant research approach to draw on here.

Throughout the study, discourse with critical friends from both insider and outsider perspectives were actively employed to provide increased reflexivity and perspective (Kember *et al.*., 1997; Noor & Shafee, 2021). Ethical Approval was gained and informed consent obtained and recorded for each participant.

To mitigate the possibility of insider researcher bias, the researcher undertook a bracketing exercise ( Tufford & Newman, 2010) whereby their own beliefs and experiences of role-modelling in GP were explicitly outlined before each interview to consciously keep them in abeyance and prevent them colouring the interview content. Further, acknowledging, documenting and reflecting upon preconceptions and insider researcher bias allowed reflexivity during the analysis of data and coding phases.

## Results

**Figure 3.**
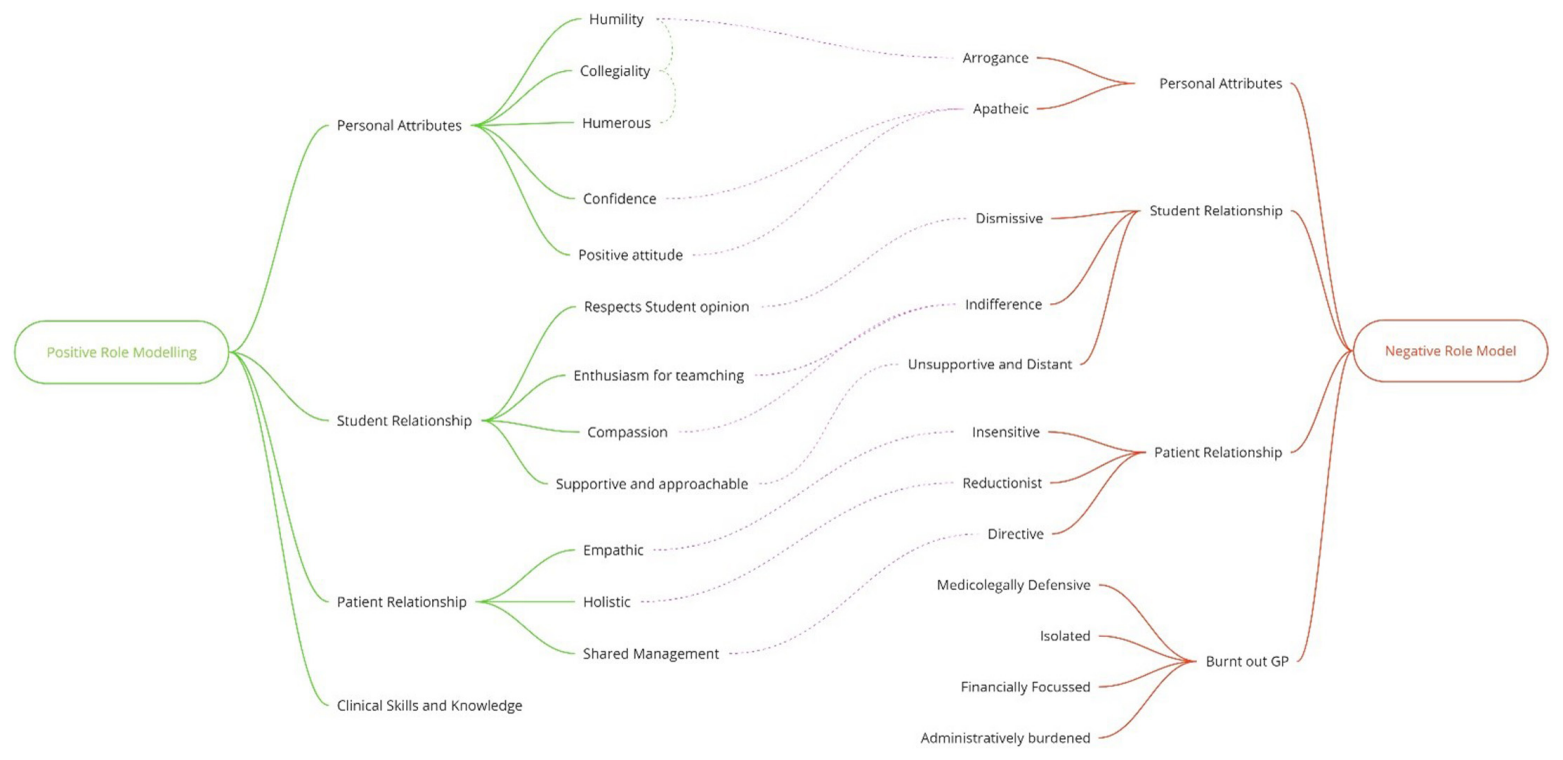
Positive & Negative Role-Model Attributes.

Analysis of data gave insights regarding student perception of positive and negative role modelling in three corresponding domains: Personal Attributes, Student Relationship and Patient Relationship. Figure 3 depicts the main emerging concepts from the interviews. 

### Positive role model attributes

 The themes depicted above are now evidenced through the students’ own words.

***Student relationship***

The interpersonal relationship that developed between the student and their mentor was commonly discussed, with students emphasising the need for mutual respect and collegiality:

*[I was treated with]* .. *open respect. That is, as a colleague, rather than their junior.*
*…a patient came in with chronic pain, a young patient .. were only in their 20s had it for five years, and I just mentioned in passing when he was doing investigations like, oh, what about HLA B-27?.....He took it as a genuine idea, It felt like we were colleagues, rather than just students at the practice,*

Interestingly, students reported compassion toward their life situations and stressors as a crucial contributor toward the role-modelling process with sharing of similar life experiences as a pivotal mechanism for this:

*...a lot of them were Warwick grads, ...and it's that almost shared understanding ... [They were like… ] .We're going to help you. We understand what we've been through....That helps that relationship. I always felt comfortable...They understood the pressures...*

The perception that the GP had a genuine enthusiasm for teaching made substantial contribution to the role modelling process.

*[My GP was] ..very proactive with teaching and kind of organization as well. So we knew exactly what to expect when we come into a session.*
*[They] really set me down beforehand and explaining, like, how to make the patient as comfortable as possible.*

Other significantly represented themes included “Giving immediate feedback” and giving students the opportunity to discuss career options.

***Patient relationships***

A unifying RM theme from students supported observation of positive GP-patient relationships encompassing empathy, rapport, kindness, shared management and holistic understanding.

*[Thet were] very interested in their patients’ lives and so very supportive. Which just makes you think that's more of the kind of doctor I want to be.*
*[They showed] empathy and understanding where the patient was coming from ... relating management specific to the patient.*

***Clinical skills and knowledge***

Students reported thorough knowledge of physiology and pathology gave them great confidence in their role model:

*His professional knowledge was excellent, and it's almost as though he was [ ] very strong. It's almost like the “by the book GP.*

***Personal attributes***

Meaningful reflection regarding the contribution of personal attributes of the GP to the role-modelling process was observed in the data. Humility, collegiality, humour and confidence, including “confidence in not knowing” were considered strong positive contributors to the process:

In discussing the importance of collegiality one student noted that:

*[My role model] seemed very friendly with the reception staff.*

Whilst another commented:

*it was really nice to see discussions between colleagues as well,*

Likewise, the concept of humility was also reported as being important:

*His attitude was that he was there to serve the patient I really, really liked (that).*

Whilst the importance of humour was commented on:

*Not about the patient and but like... Yeah, sharing a joke. And there was sort of general laughter and just I think it made the situations more comfortable.*

Perhaps, most importantly the confidence shown by role-models in not knowing the answers made an impression **:**

*After all the tests and things its idiopathic, which basically means we don't know, like, and I think the fact that there was sort of confident in that fact that they didn't know the patients felt relief because, like, OK, if the GP doesn't know then. You know it's. Yeah.*

### Negative role-model attributes

The data revealed several negative-role-model themes corresponding to those identified in positive role-models (Student Relationship, Patient Relationship and Personal Attributes) whilst also highlighting a theme unique to negative RM; “The Burnt-out GP”.

***Student relationships***

The relationship between GP and student featured highly in relation to negative role-modelling. The learners wanted the GP to be aware of and considerate to their individual circumstances such as imminent assessment deadlines and long commutes:

*We had been balancing a lot at the time. I think we had multiple assessments... being handed in the audit being one of them...and we were also commuting from really far away...a 90-mile round trip and... the GP was expecting us to come in on a Saturday to collect the data and I said I can't afford it.*

Further, students wanted validation and consideration of knowledge gaps appropriate to their stage of learning:

* ... a little bit harsh or almost a little mocking of... Elements where there was like perhaps a gap in knowledge,*
*Less appreciation for the fact that this was our first GP placement...So we were babies essentially... to get the right diagnosis or ask the right questions straight away...Wow, you should know this. And ‘it’s like, yeah, of course I should know this, but...*

One aspect of the GP-student relationship that had resonance with students was a lack of respect for student opinion:

*I feel like you can tell if you're being treated like a colleague rather than... I don't know someone else standing in the corner observing, doing nothing.*

The impression that the GP did not want students with them further contributed to the negative role-model process:

*[They were] overstressed and that meant they were less engaged with their teaching.*

***Patient relationship***

Students held the GP-Patient relationship as a core value and commonly associated negative patient relationships with negative GP role models.

*one of them made a patient cry... don't think it reflected well on his rapport with patients...lack of trust...contempt towards the patient.*
*It seemed a bit like they don’t really care anymore... He didn’t really consider mental health as an issue... and was quite abrupt and...confrontational with patients... there wasn’t much empathy*
*... they did know their patients a bit more and yeah, they probably say alright, this one coming up, he's a bit of a nightmare*.

***Personal attributes***

Certain humanistic properties were identified within the negative role model theme by students. Arrogance had its opposite of humility identified in positive role modelling and was repeatedly referenced:

*this GP was untouchable...Everyone had to know their place...Don't argue with a partner. There's no, there was no openness. There was no conversation. There's no dialogue between all. Actually, I'm not sure if they're doing this right. I think we need to discuss this. We need to come up with that. There was none of that...there was no liaising with colleagues…*

### Burnt out GP

Students revealed that GP Stress was the next most commonly associated negative role-modelling theme after student and patient relationship and personal attributes:

*It was [a] really pressured environment and I think particularly with the first GP was talking about, they were very stressed... *
*Uhm, everyone seems very stressed up with the phone consultations ...trying to go through everything and then have to get them in anyway and then you go through everything again ... I feel like the GP’s we were with seemed very stressed.*

It was evident that the students felt such stress impacted their teaching strongly.

*the GP's like Oh, you know, it's a busy list but I need my headset in because I need my hands to be free and to type and I thought at the time was like well if you have it on speaker, your hands are still free. But I didn't feel I was able to sort of make that comment because they seem quite stressed anyway.*
*…just felt like it was kind of going through the motions, you know, just doing the bare minimum to get this, like this session out the way. So, it wouldn't be kind of the engaging teaching..*

### Gender and ethnicity

Students described empowerment in association with identification of a successful role-model of similar gender age and ethnicity. Students in general did find this difficult to openly talk about perhaps reflecting the effect of a white male researcher or societal taboos discussing race related issues.

*" ...from (a), similar ethnic background to me and was a female as well, and I found she had a good amount of responsibility in the practice, and it was really empowering to sort of see her go about her day to day, manage these patients, managed her administrative responsibilities... "*

## Discussion

This study found that students on GP placement negotiate a range of social-cognitive processes relating to physical setting, professional group, and community. These cognitive processes converge to shape their views on professional identity and career pathways. A positive GP role-model, guides evolving perceptions and experiences, including creation of professional identity, character development and career choices. Students on placement learn largely from observation, imitation, and modelling or demonstrating ( Lamb *et al.*, 2022). From a social-constructivist perspective, students ‘learn their profession’ through a complex cycle of conscious and unconscious observation and subsequent reflection(s) on how to assimilate what they perceive as important observed actions into their own value systems and behaviours. Findings indicate the student’s approach to decoding role-modelling (how they make decisions on whether to mimic the behaviours and associated outlooks and values of the GP role-model) was akin to what Passi and Johnston describe as ‘Exposure Phase’ and ‘Evolution Phase’ ( Passi & Johnson, 2016b).

### Relationship to student

The GPs’ inclination and capacity to understand and relate to the medical student positionality was identified as a dominant (positive) theme in all interviews and resonates with findings of other studies ( Bazrafkan *et al.*, 2019; Griffin & Cook, 2009). Students highlighted more enjoyable, meaningful, and productive learning interactions with role-models they felt they could easily identify with. This concurred with findings from the wider literature espousing the significance of modelees identifying “possible selves” ( Burack *et al.*, 1997) as potential role-models. Similarly, the strong “near peer” effect on role-modelling by those of closer age such as GP trainees ( Barber *et al.*, 2018) chimed with our findings.

Students expressed affinity towards role-models who took the time and effort to get to know their students on an individual basis as found previously ( Jochemsen-Van Der Leeuw *et al.*, 2014), particularly tailored consideration regarding future portfolio career options.

Practical guiding advice, such the setting out realistic expectations for students was also perceived positively in alignment with education contract recommendations ( Anderson *et al.*, 2014) and discussed regarding medical students in the wider literature ( Stubbing *et al.*, 2019) but not previously in the context of RM.

***Respecting medical student opinion***

 “Respecting student opinion” emerged as a key positive role-model theme. Critically, students felt a strong sense of being on a transitional journey, to become a medical professional. Data revealed that this dynamic, helped students to be more optimistic and confident when they were forming ideas and impressions on their developing professional identity.

***Enthusiasm towards teaching***

Enthusiasm towards teaching had been identified as a positive role-model behaviour in a recent study by Mackie ( Mackie & Alberti, 2021) and the study here corroborated this observation in the post-Covid-19 context especially important given the decreased face-to-face contact time with clinicians they had experienced during periods of remote learning and disrupted clinical placements. Moreover, providing immediate, high-quality constructive feedback to students on their clinical performance was acknowledged by the respondents as a significant positive component in this role-model teaching dynamic. Timely constructive feedback has long been identified as a key teaching skill and highly desirable in clinical role-models ( Burgess *et al.*, 2015). This study adds weight to any claims of transferability of these findings (by Burgess ibid) to the contemporary GP student context.

### Relationship to patient; holistic care, shared decision making and reflecting on empathy

Much commonality was found regarding themes pertaining to patient empathy in the literature and this study ( Amalba *et al.*, 2017; Burack *et al.*, 1997). Students developed the idea that demonstration of empathy toward the patient was a highly valued behaviour constituting the positive role-model. Data strongly revealed the converse was also true. Not previously noted in the literature, the students in this study added that initiation and invitation of dialogue regarding cognisance of empathy toward the patient and barriers towards this empathy was a strongly positive role-model behaviour.

Student support for **holistic care** models in GP role-modelling student settings have been previously noted across Europe ( Lublin, 1992; Miettola *et al.*, 2005). Participants in this study supported transferability of this observation to a UK context developing themes around positive role-modelling in relation to active engagement with holistic care and negative role-modelling when single approach medical model only practice was observed.

**Shared Decision Making** (SDM) with doctors in partnership with patients has long been held as the aspirational paradigm for patient management ( Elwyn *et al.*, 2012) with observational learning of this skill previously noted ( Oerlemans *et al.*, 2021). Oerlemans (Ibid) sought to inform the extent to which observation of SDM shapes identity as a positive role-model. Participants reported observation of skilfully applied SDM processes as a strong element in the make-up of the positive role-model and that the converse was true such that plans without SDM were associated with negative role-modelling.

Participants identified cynicism and negativity toward any patient as a strongly negative RM trait agreeing with previous studies in other medical disciplines ( Wear & Skillicorn, 2009).

### Clinical skill; knowledge and professionalism

Clinical Knowledge ( Elzubeir & Rizk, 2001), familiarity with guidelines, drawing on latest research evidence, commitment to professionalism and updating knowledge ( Paice *et al.*, 2002) have been unified under the umbrella terms of “clinical skill or “patient care qualities” ( Jochemsen-Van Der Leeuw *et al.*, 2013) and were perceived as significant role-model characteristics in the general medical literature. Respondents reaffirmed these attributes as significant in marking out what makes a positive GP role-model.

### Gender and ethnicity

There were indications in this small study that the role of gender and ethnicity may be significant in role-model identification in agreement with previous findings ( Kutob *et al*., 2006). Study design to sensitively probe this area further with specific consideration to sensitivities around the ethnicity of the investigator and investigator reflexivity are warranted to inform in this significant area, especially with acknowledgement of the influence on speciality recruitment and the need to reflect U.K. population within the GP community ( Lamb *et al*., 2022).

### Strengths and limitations

The qualitative approach facilitated meaningful and deep respondent views. The co-authors of the article from different institutional and academic backgrounds provided independent reflexivity.

The small sample size of 10 graduate students from a single institution suggests caution in generalising findings. However, lack of emergence of new themes in the last two interviews indicated that data saturation was reached. Future studies utilising purposive sampling could explore the roles of ethnicity and gender identity in role-modelling. Further triangulation of data with identified positive and negative RMs could expand the range of views captured.

The interviews were undertaken by a faculty member which may have influenced participant behaviour. The Participant Information Sheet made it clear that all information was treated confidentially to help obviate this problem.

## Conclusions and recommendations

In conclusion, this study adds to the wider body of literature by exploring the influences GP role-modelling has on medical student training experience during the exceptional circumstances of a global pandemic and the immediate post- pandemic period. The findings offer unique insights into the influence and impact of GP role-modelling on medical student’s experiences and perceptions during a time of extraordinary circumstances supporting consideration of key recommendations to enhance RM (See also Extended Data).

Thematically ordered recommendations are given in the below table. Together, these recommendations may be applied when identifying suitable student placements with consideration to role-modelling

**Table:** 

Theme	Recommendation
**Student Relationship**	Defining clear, realistic expectations of the student.
	Eliciting and respecting student opinion as a young professional.
	Demonstrate teamworking highlighting understanding of difficulties faced by other team members. With positive explanations of colleague behaviour.
	Consider “Near Peer effect” when highlighting potential Role Models.
	Explain to learners why you enjoy teaching.
	Share and encourage discourse regarding ambiguity and areas of your own educational need.
	Explore and exemplify portfolio working as applied to the individual student interests.
	Frame discussions constructively around medicolegal, resource planning and financial issues.
	Offer immediate, specific constructive feedback.
	Encourage discussion regarding empathy toward patients including examples of when and why this can be difficult.
**Patient Relationship**	Model and discuss shared decision marking.
	Create appointment length to allow patient time to fully express their problem.
	Explain and model a holistic approach to patient care.
	Demonstrate commitment to updating knowledge and skills and explain what areas you find most interesting and why.
**Clinical Skill**	Demonstrate familiarity with guidelines and evidence-based medicine.

## Ethics and consent statement

This study has been reviewed and approved on behalf of the Schools of Medicine and Life Sciences Research Ethics Committee on Date 2nd December 2021 and approval number SMED REC Number 21/ 94. Written informed consent was documented on forms approved during ethics review (see Extended Data).

## Data Availability

Qualitative research data where participants did not want their transcript in entirety to be viewed as this may be identifiable despite being anonymised as descriptions of examples are given from clinical medical contexts. Anonymised datasets can be requested via email to h.sutcliffe@warwick.ac.uk on an individual basis owing to concerns over identification of the described positive or negative role-models. Successful requests should include institutional affiliation and purpose of data use. Zenodo: Learning from the best: How medical-students construct role models in general practice during the COVID-19 pandemic and what factors influence this process, a qualitative study. https://doi.org/10.5281/zenodo.13941730. Sutcliffe (2024) **This project contains the following extended data:** 1. ASME 2023 Poster Sutcliffe final.pdf 2. Consent_form .docx 3. Non-Clinical-Research-Ethics-FORM-B-Medium-High-Risk-Application-Form-v3-29032019 (002).docx 4. PIS - Participant Information Sheet.docx 5. Question Guide - Role Modelling.docx 6. Risk Assessment Dundee research.docx Data are available under the terms of the Creative Commons Attribution 4.0 International license (CC-BY 4.0)

## References

[ref-1] Amalba A Abantanga FA Scherpbier AJJA : Community-based education: the influence of role modeling on career choice and practice location. *Med Teach.* 2017;39(2):174–180. 10.1080/0142159X.2016.1246711 27841070

[ref-2] Anderson G Boud D Sampson J : Learning contracts: a practical guide. *Learning Contracts.* 2014. 10.4324/9781315041766

[ref-3] Bandura A : Social learning theory. Englewood Cliffs,1977.

[ref-4] Barber S Brettell R Perera-Salazar R : UK medical students' attitudes towards their future careers and general practice: a cross-sectional survey and qualitative analysis of an Oxford cohort. *BMC Med Educ.* 2018;18(1): 160. 10.1186/s12909-018-1197-z 29973203 PMC6030758

[ref-5] Bazrafkan L Hayat AA Tabei SZ : Clinical teachers as positive and negative role models: an explanatory sequential mixed method design. *J Med Ethics Hist Med.* 2019;12:11. 10.18502/JMEHM.V12I11.1448 32328224 PMC7166239

[ref-6] Burack JH Irby DM Carline JD : A study of medical students' specialty-choice pathways: trying on possible selves. *Acad Med.* 1997;72(6):534–541. 10.1097/00001888-199706000-00021 9200589

[ref-7] Burgess A Goulston K Oates K : Role modelling of clinical tutors: a focus group study among medical students. *BMC Med Educ.* 2015;15(1): 17. 10.1186/s12909-015-0303-8 25888826 PMC4335700

[ref-8] Charmaz K : The legacy of Anselm Strauss in constructivist grounded theory. In: *Studies in Symbolic Interaction. * 2008;32:127–141. 10.1016/S0163-2396(08)32010-9

[ref-9] Elwyn G Frosch D Thomson R : Shared decision making: a model for clinical practice. *J Gen Intern Med.* 2012;27(10):1361–1367. 10.1007/S11606-012-2077-6 22618581 PMC3445676

[ref-10] Elzubeir MA Rizk DE : Identifying characteristics that students, interns and residents look for in their role models. *Med Educ.* 2001;35(3):272–277. 11260451 10.1046/j.1365-2923.2001.00870.x

[ref-11] Flick U : An introduction to qualitative research. Sage Publications Ltd,1998. Reference Source

[ref-12] Glaser BG Strauss AL : The discovery of grounded theory. In: *The Discovery of Grounded Theory.*Aldine Publishing Co,1967;1–18. Reference Source

[ref-13] Griffin A Cook V : Acting on evaluation: twelve tips from a national conference on student evaluations. *Med Teach.* 2009;31(2):101–104. 10.1080/01421590802225788 19330668

[ref-14] Holden J Cox S Irving G : Rethinking role models in general practice. *Br J Gen Pract.* 2020;70(698):459–460. 10.3399/BJGP20X712517 32855143 PMC7449423

[ref-15] Jochemsen-Van Der Leeuw HGAR Van Dijk N Van Etten-Jamaludin FS : The attributes of the clinical trainer as a role model: a systematic review. *Acad Med.* 2013;88(1):26–34. 10.1097/ACM.0B013E318276D070 23165277

[ref-16] Jochemsen-Van Der Leeuw HGAR Van Dijk N Wieringa-De Waard M : Assessment of the clinical trainer as a role model: a Role Model Apperception Tool (RoMAT). *Acad Med.* 2014;89(4):671–677. 10.1097/ACM.0000000000000169 24556764 PMC4885572

[ref-33] Kember D Ha TS Lam BH : The diverse role of the critical friend in supporting educational action research projects. *Educational Action Research.* 1997;5(3):463–481. 10.1080/09650799700200036

[ref-17] King N : The qualitative research interview. In: *Qualitative methods in organizational research: a practical guide.*Sage Publications, Inc,1994;14–36. Reference Source

[ref-23] Kutob RM Senf JH Campos-Outcalt D : The diverse functions of role models across primary care specialties. *Fam Med.* 2006;38(4):244–51. 16586170

[ref-18] Lamb E Burford B Alberti H : The impact of role modelling on the future general practitioner workforce: a systematic review. *Educ Prim Care.* 2022;33(5):265–279. 10.1080/14739879.2022.2079097 35904161 PMC9519122

[ref-34] Lindqvist H Forsberg C : Constructivist grounded theory and educational research: constructing theories about teachers’ work when analysing relationships between codes. *International Journal of Research & Method in Education.* 2023;46(2):200–210. 10.1080/1743727X.2022.2095998

[ref-19] Lublin JR : Role modelling: a case study in general practice. *Med Educ.* 1992;26(2):116–122. 10.1111/j.1365-2923.1992.tb00136.x 1565027

[ref-20] Mackie E Alberti H : Longitudinal GP placements - inspiring tomorrow's doctors? *Educ Prim Care.* 2021;32(3):149–156. 10.1080/14739879.2020.1846142 33228461

[ref-21] Miettola J Mäntyselkä P Vaskilampi T : Doctor-patient interaction in Finnish primary health care as perceived by first year medical students. *BMC Med Educ.* 2005;5(1): 34. 10.1186/1472-6920-5-34 16162300 PMC1242232

[ref-22] NHS longterm workforce plan. 2023. Reference Source

[ref-35] Noor MSAM Shafee A : The role of critical friends in action research: a framework for design and implementation. *Practitioner Research.* 2021;3:1–33. 10.32890/pr2021.3.1

[ref-24] Oerlemans AJM Knippenberg ML Olthuis GJ : Learning shared decision-making in clinical practice. *Patient Educ Couns.* 2021;104(5):1206–1212. 10.1016/j.pec.2020.09.034 33041158

[ref-25] Paice E Heard S Moss F : How important are role models in making good doctors? *BMJ.* 2002;325(7366):707–710. 10.1136/bmj.325.7366.707 12351368 PMC1124228

[ref-26] Passi V Johnson N : The hidden process of positive doctor role modelling. *Med Teach.* 2016a;38(7):700–707. 10.3109/0142159X.2015.1087482 26524562

[ref-27] Passi V Johnson N : The impact of positive doctor role modelling. *Med Teach.* 2016b;38(11):1139–1145. 10.3109/0142159X.2016.1170780 27089216

[ref-28] Passi V Johnson S Peile E : Doctor role modelling in medical education: BEME guide no. 27. *Med Teach.* 2013;35(9):e1422–36. 10.3109/0142159X.2013.806982 23826717

[ref-29] Stubbing EA Helmich E Cleland J : Medical student views of and responses to expectations of professionalism. *Med Educ.* 2019;53(10):1025–1036. 10.1111/medu.13933 31509286

[ref-32] Sutcliffe H : Learning from the best: how medical-students construct role models in general practice during the COVID-19 pandemic and what factors influence this process, a qualitative study. *Zenodo.* 2024. 10.5281/zenodo.13685031 PMC12231363 40626106

[ref-30] Tufford L Newman P : Bracketing in qualitative research. *Qual Soc Work.* 2010;11(1):80–96. 10.1177/1473325010368316

[ref-31] Wear D Skillicorn J : Hidden in plain sight: the formal, informal, and hidden curricula of a psychiatry clerkship. *Acad Med.* 2009;84(4):451–458. 10.1097/ACM.0b013e31819a80b7 19318777

