# Supplementary material for: Learning from the best: How medical-students construct role models in general practice during the COVID-19 pandemic and what factors influence this process, a qualitative study
Source: MedEdPublish (2016). 2025 Mar 27;14:278. Originally published 2024 Nov 15. [Version 2] doi: 10.12688/mep.20594.2 (PMC12231363; doi:10.12688/mep.20594.2)
Supplement: Peer review history

# Reviewer report: Reviewer response for version 2

**DOI:** 10.21956/mep.22472.r42184

**Published:** 2025-07-28

**Authors:** Claire Stocker

**Affiliations:** 1 Aston Medical School, Aston University, Birmingham, England, UK

The submitted manuscript investigates how medical students experience and construct role models during their general practice placements, particularly in the context of the COVID-19 pandemic. The quality of the English is good, but there are several points where the manuscript requires improvement.

 The introduction is not presented with due reference to the published field, with recent studies on the roles of gender and ethnicity in role-modelling missing. Additionally, the discussion of negative role-modelling is limited, despite its potential significance in shaping students’ experiences. As such, the manuscript does not appropriately citate current literature.

 The small sample size of ten participants from a single institution makes the conclusions unreliable. While the authors claim theoretical saturation, this is not robustly demonstrated in the text. The methodological choice to exclude any quantitative triangulation, such as frequency counts or participant demographics, further limits the technical soundness of the study.

 Moreover, the description of data analysis lacks depth, particularly concerning axial coding and the execution of bracketing exercises to mitigate researcher bias. The authors do note the small sample size, the single-institution context, and the potential for researcher bias due to the insider status of the interviewer. However, they do not sufficiently explore how these limitations might have influenced the findings or how they could be mitigated in future research.

 The assertion that the study offers “unique insights” is overstated, as the findings largely corroborate existing literature rather than generating novel theoretical contributions. Moreover, the recommendations for educational practice, while practical, lack specificity in terms of actionable implementation strategies. This limits the conclusions’ impact and practical utility.

Have any limitations of the research been acknowledged?

Partly

Is the work clearly and accurately presented and does it cite the current literature?

Partly

If applicable, is the statistical analysis and its interpretation appropriate?

Not applicable

Are all the source data underlying the results available to ensure full reproducibility?

Yes

Is the study design appropriate and is the work technically sound?

Partly

Are the conclusions drawn adequately supported by the results?

Partly

Are sufficient details of methods and analysis provided to allow replication by others?

Partly

Reviewer Expertise:

Professionalism, learner support, educator support, curriculum design

I confirm that I have read this submission and believe that I have an appropriate level of expertise to confirm that it is of an acceptable scientific standard, however I have significant reservations, as outlined above.

# Reviewer report: Reviewer response for version 2

**DOI:** 10.21956/mep.22472.r42183

**Published:** 2025-07-03

**Authors:** Elizabeth Iris Lamb

**Affiliations:** 1 Newcastle University, Newcastle upon Tyne, UK

This qualitative study aimed to explore the lived experience of medical students encountering potential role models during their GP placement during the Covid-19 pandemic. The literature review and discussion of relevant theory set the scene for the study, and identifies a gap in the literature which the study aims to address. Qualitative interviews were undertaken with 10 third year medical students. Grounded theory informed the approach and subsequent analysis. Themes were separated into positive and negative role model attributes, and quotes were selected to support the themes. The themes were then related back to existing literature in the discussion section and recommendations made which seemed to be aimed at the GP role model.

 Though the title mentions the pandemic, there is then little further information about the impact of the pandemic on medical education within the paper, the quotes do not make reference to the pandemic, and the discussion section does not consider how role modelling may have been impacted by the pandemic. Presumably it did impact on the role modelling process as teaching and consulting took place remotely? How did mask wearing/ social distancing affect the process? The recommendations focus on actions for the role model, but these could be improved through recommendations for educational institutions- eg to support medical students to identify role models of similar gender, age and ethnicity.

Have any limitations of the research been acknowledged?

Yes

Is the work clearly and accurately presented and does it cite the current literature?

Yes

If applicable, is the statistical analysis and its interpretation appropriate?

Not applicable

Are all the source data underlying the results available to ensure full reproducibility?

No

Is the study design appropriate and is the work technically sound?

Partly

Are the conclusions drawn adequately supported by the results?

Partly

Are sufficient details of methods and analysis provided to allow replication by others?

No

Reviewer Expertise:

Realist research and systematic review, in the area of role modelling in medical education

I confirm that I have read this submission and believe that I have an appropriate level of expertise to confirm that it is of an acceptable scientific standard, however I have significant reservations, as outlined above.

# Reviewer report: Reviewer response for version 2

**DOI:** 10.21956/mep.22472.r42181

**Published:** 2025-07-03

**Authors:** Anne Simmenroth

**Affiliations:** 1 University Hospital Würzburg, Würzburg, Germany

This are valuable results which should be indexed, esp. die “negative role models” –topics that we normally do not like to discuss in teaching! For me, there are still some unclear aspects of recruitment and data collection (see comments to authors).

**Abstract**

 Methods: Please add, if you did individual or group interviews/focus groups.

**Introduction:**

 The introduction is well written, cites the relevant literature. Could you add one more sentence on why this methodology was chosen?

**Methods:**

 In the methods-section many details remain unclear:

 How were the 10 students selected? Was attention paid to contrast (e.g. age, gender, social origin)? Was it a group discussion or individual interviews? If group: how many? Who moderated/conducted the interviews/the group? Where the questions piloted? (if not, please add it to the limitations). What was the opening question to stimulate the narrative? Who was present, how were the interviews moderated?  When exactly was the data collected? How long did the interviews last? Why only 10 participants - or were more planned at the beginning and saturation was reached with the 10th participant?  Why only 3rd year and not older students?

 Please briefly describe in which year the medical students at your faculty have a rotations in general practice/family medicine and for how long – readers from outside the UK  cannot know this.

 What about data management and data protection?

**Results**

 I really like the results section, as there are lots of quotes. Please add a short table with some information about the students you interviewed: age, gender, social origin, medical related training before studying.

**Discussion**

 Please start the discussion with a short summary about your methods and 1-2 main results.

 And add in limitations: why did you not you choose focus groups? With group discussions, more data can be collected (economically) and different insights can be gained in homogeneous or inhomogeneous  groups than in individual interviews. Please mention this briefly in the limitations.

**Figures/Graphs**

 A heading is missing from the table.

Have any limitations of the research been acknowledged?

Partly

Is the work clearly and accurately presented and does it cite the current literature?

Yes

If applicable, is the statistical analysis and its interpretation appropriate?

Not applicable

Are all the source data underlying the results available to ensure full reproducibility?

No source data required

Is the study design appropriate and is the work technically sound?

Partly

Are the conclusions drawn adequately supported by the results?

Yes

Are sufficient details of methods and analysis provided to allow replication by others?

No

Reviewer Expertise:

teaching communication skills, social skills, teaching prevention, motivational interviewing, OSCE, didactics, mental illness in primary care.

I confirm that I have read this submission and believe that I have an appropriate level of expertise to confirm that it is of an acceptable scientific standard, however I have significant reservations, as outlined above.

# Reviewer report: Reviewer response for version 2

**DOI:** 10.21956/mep.22472.r41278

**Published:** 2025-03-29

**Authors:** Birgitte Schoenmakers

**Affiliations:** 1 KU Leuven, Leuven, Belgium

Thank you for the revision, I agree to indexing.

Have any limitations of the research been acknowledged?

Partly

Is the work clearly and accurately presented and does it cite the current literature?

Yes

If applicable, is the statistical analysis and its interpretation appropriate?

Partly

Are all the source data underlying the results available to ensure full reproducibility?

Yes

Is the study design appropriate and is the work technically sound?

Partly

Are the conclusions drawn adequately supported by the results?

Partly

Are sufficient details of methods and analysis provided to allow replication by others?

Yes

Reviewer Expertise:

curriculum development

I confirm that I have read this submission and believe that I have an appropriate level of expertise to confirm that it is of an acceptable scientific standard.

# Reviewer report: Reviewer response for version 1

**DOI:** 10.21956/mep.22043.r40833

**Published:** 2025-01-13

**Authors:** Birgitte Schoenmakers

**Affiliations:** 1 KU Leuven, Leuven, Belgium

Overall, the article provides valuable insights into the role-modelling process in medical education during the COVID-19 pandemic. But, I have some concerns:

- The small sample size (10 students from a single institution) limits the generalizability of the findings. The study does not deeply explore how factors like ethnicity and gender identity influence role-modelling experiences. The study also emphasizes positive role-modelling but could benefit from a more balanced exploration of negative role-modelling and its impacts. Finally, interviews conducted by a faculty member might influence participant responses despite measures to ensure confidentiality.
- From a methodological point of view, I would suggest integrating additional theories related to role-modelling and medical education to strengthen the theoretical framework.
- Overall, I also miss a more comprehensive approach by incorporating quantitative data alongside qualitative interviews.

In conclusion, providing more detailed and specific recommendations for implementing the study’s findings in medical education curricula can increase the practical utility of the research.

Have any limitations of the research been acknowledged?

Partly

Is the work clearly and accurately presented and does it cite the current literature?

Yes

If applicable, is the statistical analysis and its interpretation appropriate?

Partly

Are all the source data underlying the results available to ensure full reproducibility?

Yes

Is the study design appropriate and is the work technically sound?

Partly

Are the conclusions drawn adequately supported by the results?

Partly

Are sufficient details of methods and analysis provided to allow replication by others?

Yes

Reviewer Expertise:

curriculum development

I confirm that I have read this submission and believe that I have an appropriate level of expertise to confirm that it is of an acceptable scientific standard, however I have significant reservations, as outlined above.

# Reviewer report: Reviewer response for version 1

**DOI:** 10.21956/mep.22043.r40567

**Published:** 2025-01-06

**Authors:** Charlotte Hespe

**Affiliations:** 1 University of Notre Dame, Darlinghurst, Australia

Thank you for the opportunity to provide feedback on this well written and interesting qualitative research article looking at constructs of Role models in GP for medical students. The article is well constructed and easy to read. However the author states they used Constructivist Grounded Theory to generate a new theory and that the data was analysed using theoretical and axial coding. There were only 10 interviews conducted, and this was justified by saying the final two interviews did not provide any new material. The author also mentions undertaking a bracketing exercise prior to each interview to mitigate insider researcher bias. As a reviewer I do not feel that there is adequate justification or explanation provided around any of these 3 approaches nor are they adequately documented in the results or discussion. I did not feel there was a new theory generated (in fact it fell like the interviews confirmed prior theories) and there is total lack of detail around what the bracketing exercise entailed and how the author felt it achieved the goal of preventing personal biases colouring the analysis (and interview questions). More detail and justification needs to be provided in the methods along with much stronger justification for only conducting 10 interviews. The limitations of a single Uni, only 10 interviews and a staff member conducting the interviews become overwhelmingly negative as a reader. This is unfortunate, especially when the topic and findings are so relevant to understanding the power of role models in this area of medical student experience.

Have any limitations of the research been acknowledged?

Yes

Is the work clearly and accurately presented and does it cite the current literature?

Yes

If applicable, is the statistical analysis and its interpretation appropriate?

Not applicable

Are all the source data underlying the results available to ensure full reproducibility?

Partly

Is the study design appropriate and is the work technically sound?

Partly

Are the conclusions drawn adequately supported by the results?

Yes

Are sufficient details of methods and analysis provided to allow replication by others?

Partly

Reviewer Expertise:

General Practice (clinical and academic), Medical Education, Qualtitative (and mixed methods) research in primary care

I confirm that I have read this submission and believe that I have an appropriate level of expertise to confirm that it is of an acceptable scientific standard, however I have significant reservations, as outlined above.
